# The prevalence and contributing risk factors of coronavirus disease 2019 infection in patients with metabolic syndrome

**DOI:** 10.1186/s12902-023-01351-0

**Published:** 2023-05-04

**Authors:** Zahra Bagheri-Hosseinabadi, Fatemeh Moadab, Ali Amiri, Mitra Abbasifard

**Affiliations:** 1 Molecular Medicine Research Center, Research Institute of Basic Medical Sciences, Rafsanjan University of Medical Sciences, Rafsanjan, Iran; 2 Department of Clinical Biochemistry, School of Medicine, Rafsanjan University of Medical Sciences, Rafsanjan, Iran; 3 Student Research Committee, Rafsanjan University of Medical Sciences, Rafsanjan, Iran; 4 Immunology of Infectious Diseases Research Center, Research Institute of Basic Medical Sciences, Rafsanjan University of Medical Sciences, Rafsanjan, Iran; 5 Department of Orthodontics, College of Stomatology, The First Affiliated Stomatological Hospital, Xi’an Jiaotong University, Xi’an, 710004 PR China; 6 Department of Internal Medicine, Ali-Ibn Abi-Talib Hospital, School of Medicine, Rafsanjan University of Medical Sciences, Rafsanjan, Iran

**Keywords:** Metabolic syndrome, Severe acute respiratory syndrome coronavirus 2, Coronavirus disease 2019, Infection susceptibility

## Abstract

**Background:**

Components of metabolic syndrome (MetS) was reported to contribute to severe and worse outcomes of coronavirus disease 2019 (COVID-19). Hereby, we evaluated the association of MetS and its components with susceptibility to COVID-19.

**Methods:**

Here, 1000 subjects with MetS were recruited that were diagnosed via the International Diabetes Federation (IDF) criterion. Real-time PCR was exerted to detect SARS-CoV-2 in the nasopharyngeal swabs.

**Results:**

Among the MetS patients, 206 (20.6%) cases were detected to have COVID-19. Smoking (OR = 5.04, 95%CI = 3.53–7.21, *P* < 0.0001) and CVD (OR = 1.62, 95%CI = 1.09–2.40, *P* = 0.015) were associated with increased chance of COVID-19 infection in the MetS patients. BMI was significantly higher (*P* = 0.0001) in MetS cases with COVID-19 than those without COVID-19. Obesity was associated with increased susceptibility to COVID-19 in MetS patients (OR = 2.00, 95%CI = 1.47–2.74, *P* < 0.0001). Total cholesterol, TG, LDL were significantly higher in the MetS cases with COVID-19 than those without COVID-19. Dyslipidemia was associated with increased chance of COVID-19 (OR = 1.50, 95%CI = 1.10–2.05, *P* = 0.0104). FBS level was significantly higher in the MetS cases with COVID-19. T2DM was associated with increased risk of COVID-19 in MetS patients (OR = 1.43, 95%CI = 1.01-2.00, *P* = 0.0384). Hypertension was associated with increased chance of COVID-19 in the MetS patients (OR = 1.44, 95%CI = 1.05–1.98, *P* = 0.0234).

**Conclusions:**

MetS and its components, like obesity, diabetes, dyslipidemia, cardiovascular complications were associated with increased chance of COVID-19 infection development and probably with aggravated symptoms in such patients.

## Introduction

Coronavirus disease 2019 (COVID-19), which is caused by novel Severe acute respiratory syndrome coronavirus 2 (SARS-CoV‐2), has become a global health challenge and causes a wide range of clinical complications and, in the more intense and severe conditions, is manifested as acute respiratory distress syndrome (ARDS). The most common clinical presentation of COVID-19 are dry cough, fatigue, sore throat, and fever [1–3]. By its spike protein, SARS-CoV-2 binds to angiotensin-converting enzyme 2 (ACE2) receptor, particularly expressed on the cells from respiratory system [4–6]. COVID-19 has disseminated quickly worldwide and has conferred several health and economic problems. In March 2020, The World Health Organization (WHO) announced COVID-19 infection as a global pandemic [7]. The intense forms of COVID-19 may causes severe pneumonia that necessitates supportive cares like mechanical ventilation in hospitals [8, 9]. Aberrant activation of the immune system accompanied by higher levels of proinflammatory cytokines and chemokines is frequently reported in the intense forms of COVID-19 infection [10, 11], and the development of cytokine release syndrome associates with the worsening of the disease that might promote sepsis and multiorgan dysfunction [12–14].

The clinical course of the COVID-19 infection and the clinical symptoms range from asymptomatic conditions to intense settings of life-threatening respiratory failure that may cause mortality [15]. Several studies in different populations have revealed a number of risk factors in association with increased disease severity and mortality rate. It was shown that hypertension, obesity, chronic liver diseases, cardiovascular diseases (CVD), and diabetics are co-morbidities that increase the mortality rate and intensify the disease course in COVID-19 patients [16]. A systematic review and meta-analysis on 28 studies containing 12,995 COVID-19 patients indicated that dyslipidemia was associated with higher disease severity and mortality of COVID-19 [17]. Another meta-analysis on 203 studies involving 24,032,712 subjects demonstrated that pre-existing CVD was associated with adverse outcomes in the patients with COVID-19 [18]. Meta-analyzing the results from 46 studies containing 625,153 subjects indicated that obesity was associated 2.73-fold increased risk of COVID-19 infection, 1.72-fold higher risk of hospitalization due to COVID-19, and 1.61-fold higher COVID-19-associated mortality rate [19].

The underlying pathophysiological characteristics of metabolic syndrome (MetS) is regarded as a common representator to COVID-19 related comorbidities. This is because MetS is characterized by abdominal obesity, insulin resistance, dyslipidemia, and hypertension [20–23]. In this context, the association between MetS and related co-morbidities might exacerbate COVID-19 disease course or increase susceptibility to develop COVID-19 infection. With respect to the prevalence of MetS in various ethnicities and populations, the components of MetS might be critical risk factor for the COVID-19 infection worldwide. As a result, here we attempted, for the first time, to determine the prevalence of COVID-19 and associating risk factors in Iranian cases with MetS and divulge its relationship with development, worsening, and co-morbidities related to infection by SARS-CoV-2.

## Patients and methods

### Study subjects

In this prospective study, we enrolled 1000 subjects (with Iranian ethnicity) referred to the clinics of the Ali Ibn Abi Talib Hospital, Rafsanjan University of Medical Sciences, Kerman, Iran during June 2020 to December 2021. Detection of MetS was accomplished based on the IDF criteria [24]. According to the International Diabetes Federation (IDF), MetS was defined as having central obesity and at least two criteria such as; increased triglyceride (TG) (≥ 150 mg/dL), decreased high-density lipoprotein (HDL) cholesterol (< 40 mg/dL in men and < 50 mg/dL in women), hypertension (systolic blood pressure (BP) ≥ 130 or diastolic BP ≥ 85 mm Hg) or pre-known hypertension and fasting blood sugar (FBS) ≥ 100 mg/dL or previous history of type 2 diabetes [20, 21]. Subjects were contacted 6 months upon enrollment to be assessed for infection by SARS-CoV-2, which was determined via Real-time PCR analysis of samples from nasopharyngeal swab, computed tomography (CT) scan of chest, and clinical presentations. Patients with genetic dysfunctions, infectious diseases, immunodeficiencies, cancers, pregnancy, breastfeeding, and other metabolic and endocrine disorders were excluded from the study. None of the subjects had other complications or chronic conditions like autoimmunity, cancer, liver disease, etc. Sampling was conducted at the time of COVID-19 detection or at the end of 6th month upon enrollment. Prior to collecting 5 ml of the peripheral blood samples for detection of biochemical components, all cases voluntarily signed a written consent to participate in the study. The protocol of the undergoing investigation was endorsed by the Ethics Committee of Rafsanjan University of Medical Sciences.

### Clinical evaluations

Waist circumference (WC) was measured on a horizontal plane at the midpoint between the lower rib margin and the iliac crest. Based on IDF, a WC less than 80 cm suggests a reduced risk of type 2 diabetes, hypertension or coronary heart disease [24]. Systolic and diastolic BP (mmHg) were measured twice by a standard mercury sphygmomanometer after resting seated for 5 min. BP ≥ 130 mmHg for systolic pressure or ≥ 85 mmHg for diastolic pressure or current hypertension treatment was described as hypertension. In addition, standing height measurement was conducted with a calibrated standard wall-mounted stadiometer, following the recommendations of the World Health Organization (WHO) [25]. Weight (kg) was measured with the subjects dressed in light clothing and barefoot after overnight fasting with a standard scale. Body mass index (BMI) was measured by weight (kg) divided by height squared (m^2^). According to the WHO criteria, BMI value ≤ 18.5 kg/m2 was considered underweight, 18.5–24.9 kg/m2 as normal, 25-29.9 kg/m2 as overweight and ≥ 30 indicated obesity [25].

### Biochemical indices

Subsequent to and overnight fasting, the serum levels of total cholesterol, TG, HDL, low-density lipoprotein (LDL), liver enzymes, creatinine, blood urine nitrogen (BUN), and FBS were determined exerting clinical biochemistry autoanalyzer BT3000 Plus (Biotecnica Instruments SPA, Italy) using commercial reagents (Pars Azmoon, Iran). Blood leukocytes were enumerated using Sysmex KX-21 hematology analyzer. ESR was measured using the automated kineticphotometric method (Automatic ESR analyzer, XC-A30, Caretium Medical Instruments, China). Inflammatory indices like C-reactive protein (CRP) were measured by the Enzyme linked immunosorbent assay (ELISA) using commercial kits reagents (Pars Azmoon, Iran) and microplate reader (Stat Fax 4200, Awareness Technology Inc., UAS) Blood oxygen saturation was measured using fingertip Pulse Oximeter (Beurer PO 80, France).

### Statistical analysis

The Statistical Package for the Social Sciences (SPSS) software for windows v. 23 (SPSS, Chicago, IL, USA) was exerted for data analysis. The normality of the numerical variables was determined by the Kolmogorov–Smirnov test. The independent sample *t*-test was exerted to compare data with a normal distribution. In contrast, the non-normally distributed variables were analyzed via Mann-Whitney U test. The strength of association between nominal data with risk of COVID-19 in MetS patients was determined via calculating the odds ratio (OR) and 95% confidence interval (CI). Data presentation was conducted by mean ± standard deviation (SD) and *P* values less than 0.05 were assumed as statistically significant.

## Results

### Demographics and characterization of the study subjects

The clinical presentations and demographic data of the MetS patients are demonstrated in Table 1. Study group was comprised of 1000 MetS subjects, containing 466 (46.6%) males and 548 (53.4%) females. Among the MetS patients, 206 (20.6%) were detected to have COVID-19 infection.

**Table 1:** Demographics and clinical presentations of the MetS subjects

Characteristic	MetS patients (N = 1000)
Gender; Male/ Female (N, %)	466 (46.6%)/ 548 (53.4%)
COVID-19 cases; Yes/No (N, %)	206 (20.6%)/794 (79.4%)
COVID-19 severity; mild/severe (N, %)	189 (91.8%)/ 17 (8.2%)
Smoker/ Non-smoker	484 (48.4%)/516 (51.6%)
Familial history of CVD (Yes/No)	149 (14.9%)/ 851 (85.1%)
Age (Year, mean ± SD)	51.1 ± 10.9
Duration of MetS (Year)	14.45 ± 8.7
Duration of COVID-19 (Day)	12.5 ± 2.2
Oxygen saturation (in COVID-19 cases)	93.5 ± 5.6
Systolic BP (mmHg)	130.8 ± 22.9
Diastolic BP (mmHg)	73.3 ± 7.1
WBC (cells/mm^3^)	7378 ± 1814.1
Lymphocyte-total leukocyte ratio	26.8 ± 14.3
Neutrophil-lymphocyte ratio	10.1 ± 12.3
ALP (IU/L)	215.8 ± 44.3
AST (IU/L)	26.1 ± 7.4
ALT (IU/L)	33.1 ± 8.7
LDH (IU/L)	324.8 ± 79.5
CRP (mg/L)	3.5 ± 1.3
ESR (mm/h)	14.8 ± 10.3
BMI (kg/m^2^)	29.4 ± 5.2
WC-Male (cm)	99.6 ± 14.8
WC-Female (cm)	94.8 ± 13.4
Total cholesterol (mg/dl)	201.7 ± 36.9
TG (mg/dl)	157.1 ± 57.1
LDL (mg/dl)	128.5 ± 37.9
HDL (mg/dl)	49.1 ± 14.1
Creatinine (mg/dl)	1.61 ± 0.39
BUN (mg/dl)	22.7 ± 16.2
FBS (mg/dl)	96.2 ± 28.3
Fever (in COVID-19 cases)	200 (97.1%)
Cough (in COVID-19 cases)	197 (95.6%)
Dyspnea (in COVID-19 cases)	202 (98.1%)
Sputum (in COVID-19 cases)	122 (59.2%)
Vomiting/diarrhea (in COVID-19 cases)	118 (57.3%)
Methylprednisolone use (in COVID-19 cases)	160 (77.6%)
Dexamethasone use (in COVID-19 cases)	163 (79.1%)
Remdesivir use (in COVID-19 cases)	99 (48.1%)
Tocilizumab use (in COVID-19 cases)	4 (1.9%)
Cefixime use (in COVID-19 cases)	36 (17.5%)
Azithromycin use (in COVID-19 cases)	24 (11.7%)

MetS, Metabolic syndrome; COVID-19, Coronavirus disease 2019; WBC, White blood cell; CRP, C-reactive protein; ALP, Alkaline phosphatase; AST, Aspartate aminotransferase; ALT, Alanine aminotransferase; LDH, Lactate dehydrogenase; ESR, Erythrocyte sedimentation rate; CVD, Cardiovascular diseases; BMI, Body mass index; WC, Waist circumference; FBS, Fasting blood sugar; TG, Triglyceride; LDL, Low density lipoprotein; HDL, High density lipoprotein; BUN, Blood urea nitrogen; OR, Odds ratio; CI, Confidence interval; SD, Standard deviation; BP, Blood pressure

### Comparison of MetS patients with and without COVID-19

#### Characterization of the subjects

Table 2 demonstrates the data of the MetS patients with and without COVID-19 infection. In the MetS cases with COVID-19, 160 (77.6%) of cases were smoker, while 324 (40.8%) cases having MetS without COVID infection were smokers. It was detected that smoking was associated with increased chance of COVID-19 infection in the MetS patients (OR = 5.04, 95%CI = 3.53–7.21, *P* < 0.0001). It was observed that 41 (19.9%) of cases with MetS and COVID-19 infection had familial history of CVD, while 108 (13.6%) cases had familial history of CVD in the MetS patients without COVID-19 infection. It was seen that familial history of CVDs was associated with increased susceptibility to COVID-19 infection in the MetS patients (OR = 1.57, 95%CI: 1.06–2.34, *P* = 0.0245). No statistically significant difference was detected in the duration of MetS between two groups (*P* = 0.32).

**Table 2:** Comparison of the MetS cases with and without COVID-19 infection and related association analyses

Characteristic	MetS patients with COVID-19 (N = 206)	MetS patients without COVID-19 (N = 794)	OR (95% CI)	*P* value
Gender; Male/ Female (N, %)	88 (42.8%)/ 118 (57.2%)	378 (47.6%)/ 416 (52.4%)	0.82 (0.60–1.11)	0.2118
Smoker/ Non-smoker	160 (77.6%)/46 (22.4%)	324 (40.8%)/ 470 (59.2%)	5.04 (3.53–7.21)	< 0.0001
Familial history of CVD (Yes/No)	41 (19.9%)/ 165 (80.1%)	108 (13.6%)/ 686 (86.4%)	1.57 (1.06–2.34)	0.0245
Age (Year, mean ± SD)	51.5 ± 11.8	50.7 ± 10.2	-	0.8480
Duration of MetS (Year)	14.8 ± 8.5	14.1 ± 8.9	-	0.3240
Systolic BP	133.4 ± 21.4	128.3 ± 24.5	-	0.0018
Diastolic BP	74.2 ± 6.7	72.4 ± 7.3	-	0.0014
WBC; cells/mm^3^	9581.6 ± 2219.2	5174.4 ± 1408.9	-	< 0.0001
Lymphocyte-total leukocyte ratio	24.3 ± 14.5	29.3 ± 14.1	-	< 0.0001
Neutrophil-lymphocyte ratio	8.4 ± 10.2	11.8 ± 14.5	-	0.0016
ALP (IU/L)	318.9 ± 50.1	112.7 ± 38.6	-	< 0.0001
AST (IU/L)	31.5 ± 9.4	20.5 ± 5.4	-	< 0.0001
ALT (IU/L)	40.3 ± 10.6	25.7 ± 6.9	-	< 0.0001
LDH (IU/L)	439.5 ± 93.2	210.2 ± 65.8	-	< 0.0001
CRP (mg/L)	5.7 ± 2.1	1.4 ± 0.5	-	< 0.0001
ESR (mm/h)	21.3 ± 14.9	8.4 ± 5.8	-	< 0.0001
BMI (kg/m^2^)	31.4 ± 5.6	27.5 ± 4.7	-	0.0001
WC-Male (cm)	101.8 ± 15.5	97.4 ± 14.1	-	0.0001
WC-Female (cm)	96.2 ± 13.7	93.4 ± 13.1	-	0.0069
Total cholesterol (mg/dl)	204.9 ± 38.7	198.6 ± 35.1	-	0.0249
TG (mg/dl)	164.3 ± 58.2	149.8 ± 55.8	-	0.0010
LDL (mg/dl)	135.4 ± 39.5	121.6 ± 36.4	-	0.0001
HDL (mg/dl)	48.7 ± 12.1	49.5 ± 16.2	-	0.5079
Creatinine (mg/dl)	1.80 ± 0.5	1.4 ± 0.29	-	0.0001
BUN	25.4 ± 8.9	20.1 ± 7.3	-	0.0001
FBS (mg/dl)	98.6 ± 29.5	93.8 ± 27.2	-	0.0268

MetS, Metabolic syndrome; COVID-19, Coronavirus disease 2019; WBC, White blood cell; CRP, C-reactive protein; ALP, Alkaline phosphatase; AST, Aspartate aminotransferase; ALT, Alanine aminotransferase; LDH, Lactate dehydrogenase; ESR, Erythrocyte sedimentation rate; CVD, Cardiovascular diseases; BMI, Body mass index; WC, Waist circumference; FBS, Fasting blood sugar; TG, Triglyceride; LDL, Low density lipoprotein; HDL, High density lipoprotein; BUN, Blood urea nitrogen; OR, Odds ratio; CI, Confidence interval; SD, Standard deviation; BP, Blood pressure

#### Blood pressure

It was detected that Systolic BP was significantly higher (*P* = 0.0018) in MetS patients with COVID-19 infection (133.4 ± 21.4 mmHg) compared with those without COVID-19 infection (128.3 ± 24.5 mmHg). Moreover, diastolic BP was significantly higher (*P* = 0.0014 mmHg) in MetS cases with COVID-19 (74.2 ± 6.7 mmHg) in relation to MetS cases without COVID-19 infection (72.4 ± 7.3) (Table 2).

#### Blood leukocytes and inflammatory indices

Total number of leukocytes was observed to be significantly higher (*P* < 0.0001) in MetS cases with COVID-19 (9581.6 ± 2219.2 cells/mm^3^) in relation to MetS cases without COVID-19 infection (5174.4 ± 1408.9 cells/mm^3^). It was seen that lymphocyte-total leukocyte ratio was significantly decreased (*P* < 0.0001) in MetS cases with COVID-19 (24.3 ± 14.5) in relation to MetS cases without COVID-19 infection (29.3 ± 14.1). However, the neutrophil-lymphocyte ratio was significantly increased (*P* = 0.0016) in MetS cases with COVID-19 (11.8 ± 14.5) in relation to MetS cases without COVID-19 infection (8.4 ± 10.2).

CRP level was significantly higher (*P* < 0.0001) in MetS cases with COVID-19 (5.7 ± 2.1 mg/L) in relation to MetS cases without COVID-19 infection (1.4 ± 0.5 mg/L). Additionally, ESR level was significantly higher (*P* < 0.0001) in MetS cases with COVID-19 (21.3 ± 14.9 mm/h) in relation to MetS cases without COVID-19 infection (8.4 ± 5.8 mm/h) (Table 2).

#### Liver enzymes

It was observed that there was a significant increase (*P* < 0.0001) in ALP level in the MetS cases with COVID-19 (318.9 ± 50.1 IU/L) in relation to MetS cases without COVID-19 infection (112.7 ± 38.6 IU/L). A statistically significant increase (*P* < 0.0001) was seen in the AST level in the MetS cases with COVID-19 (31.5 ± 9.4 IU/L) compared to MetS cases without COVID-19 infection (20.5 ± 5.4 IU/L). ALT level was significantly higher (*P* < 0.0001) in the MetS cases with COVID-19 (40.3 ± 10.6 IU/L) compared to MetS cases without COVID-19 infection (25.7 ± 6.9 IU/L). It was detected that LDH level was significantly higher (*P* < 0.0001) in the MetS cases with COVID-19 (439.5 ± 93.2 IU/L) in comparison to MetS cases without COVID-19 infection (210.2 ± 65.8 IU/L) (Table 2).

#### MetS components

BMI was seen to be significantly higher (*P* = 0.0001) in the MetS cases with COVID-19 (31.4 ± 5.6 kg/m^2^) in comparison to MetS cases without COVID-19 infection (27.5 ± 4.7 kg/m^2^). It was observed that WC in both male and female subjects was significantly higher (*P* = 0.0001 and 0.0069, respectively) in the MetS cases with COVID-19 compared to MetS cases without COVID-19 infection. Total cholesterol was seen to be significantly higher (*P* = 0.0249) in the MetS cases with COVID-19 (204.9 ± 38.7 mg/dl) in comparison to MetS cases without COVID-19 infection (198.6 ± 35.1 mg/dl). TG level was significantly higher (*P* = 0.001) in the MetS cases with COVID-19 (164.3 ± 58.2 mg/dl) compared with MetS cases without COVID-19 infection (149.8 ± 55.8 mg/dl). It was detected that LDL level was significantly higher (*P* = 0.0001) in MetS cases with COVID-19 (135.4 ± 39.5 mg/dl) compared with MetS cases without COVID-19 infection (121.6 ± 36.4 mg/dl). It was detected that FBS level was significantly higher (*P* = 0.0268) in the MetS cases with COVID-19 (98.6 ± 29.5 mg/dl) compared with MetS cases without COVID-19 infection (93.8 ± 27.2 mg/dl). No statistically significant difference was detected in the HDL level between MetS cases with COVID-19 compared with MetS cases without COVID-19 infection (Table 2).

#### Co-morbidities

Obesity was seen in 98 (47.6%) cases with MetS and COVID-19, while 247 (31.1%) cases had obesity in MetS patients without COVID-19. Hence, obesity was associated with increased susceptibility to COVID-19 infection in patients with MetS (OR = 2.00, 95%CI = 1.47–2.74, *P* < 0.0001). CVD was observed in 43 (20.1%) cases with MetS and COVID-19 and 111 (13.9%) subjects having MetS without COVID-19. Therefore, CVD was associated with increased risk of COVID-19 infection in MetS patients (OR = 1.62, 95%CI = 1.09–2.40, *P* = 0.015). Type 2 diabetes mellitus (T2DM) was detected in 63 (30.5%) cases with MetS and COVID-19 and 187 (23.5%) patients having MetS without COVID-19. Presence of T2DM was associated with increased risk of COVID-19 infection in MetS patients (OR = 1.43, 95%CI = 1.01-2.00, *P* = 0.0384). It was seen that 88 (42.7%) MetS patients with COVID-19 had dyslipidemia, while 263 (33.1%) MetS patients has dyslipidemia. Hence, the analysis showed that dyslipidemia was associated with increased chance of COVID-19 infection (OR = 1.50, 95%CI = 1.10–2.05, *P* = 0.0104). Hypertension was observed in 81 (39.3%) cases and 246 (30.1%) cases in MetS patients with and without COVID-19 infection, respectively. Hypertension was observed to increase the chance of COVID-19 infection in the MetS patients (OR = 1.44, 95%CI = 1.05–1.98, *P* = 0.0234). It was seen that 23 (11.2%) cases in MetS patients with COVID-19 and 60 (7.5%) subjects in the MetS patients without COVID-19, respectively, had asthma. Hence, having asthma in the MetS patients significantly increased the chance of COVID-19 infection (OR = 1.84, 95%CI = 1.13–2.90, *P* = 0.0129). Lung diseases was seen in 66 (32.1%) MetS patients with COVID-19 and 116 (14.6%) MetS patients without COVID-19. It was detected that lung diseases significantly increased COVID-19 susceptibility in the MetS patients (OR = 2.75, 95%CI = 1.93–3.92, *P* < 0.0001) (Table 3).

**Table 3:** Association of Co-morbidities in MetS patients with risk of COVID-19.

Characteristic	MetS patients with COVID-19 (N = 206)	MetS patients without COVID-19 (N = 794)	OR (95% CI)	*P* value
Obesity	98 (47.6%)/108 (52.4%)	247 (31.1%)/ 547 (68.9%)	2.00 (1.47–2.74)	< 0.0001
CVD	43 (20.1%)/ 163 (79.9%)	111 (13.9%)/ 683 (86.1%)	1.62 (1.09–2.40)	0.0153
T2DM	63 (30.5%)/ 143 (69.5%)	187 (23.5%)/ 607 (76.5%)	1.43 (1.01-2.00)	0.0384
Dyslipidemia	88 (42.7%)/ 118 (57.3%)	263 (33.1%)/ 531 (66.9%)	1.50 (1.10–2.05)	0.0104
Neurodegenerative diseases	5 (2.4%)/ 201 (97.6%)	16 (2.1%)/ 778 (97.9%)	1.20 (0.43–3.34)	0.7136
Kidney diseases	24 (11.6%)/ 182 (88.4%)	63 (7.9%)/ 731 (92.1%)	1.53 (0.93–2.51)	0.0937
Hypothyroidism	13 (6.3%)/ 193 (93.7%)	48 (6.1%)/ 746 (93.9%)	1.04 (0.55–1.97)	0.8873
Hypertension	81 (39.3%)/ 125 (60.7%)	246 (30.1%)/ 548 (69.9%)	1.44 (1.05–1.98)	0.0234
Asthma	23 (11.2%)/ 179 (88.8%)	60 (7.5%)/ 734 (92.5%)	1.84 (1.13–2.90)	0.0129
Lung diseases	66 (32.1%)/ 140 (67.9%)	116 (14.6%)/ 678 (85.4%)	2.75 (1.93–3.92)	< 0.0001

MetS, Metabolic syndrome; COVID-19, Coronavirus disease 2019; CVD, Cardiovascular diseases; OR, Odds ratio; CI, Confidence interval; SD, Standard deviation; T2DM; Type 2 diabetes mellitus

## Discussion

Recent studies showed that comorbidities such as diabetes, hypertension, obesity, sex hormones, hyperinflammation, and immune-thrombosis contribute to severe and worse outcomes of COVID-19, suggesting that MetS and its components have association with increased chance of COVID-19 infection.

According to a cohort study, a number of co-morbidities like hypertension and diabetes existed in approximately 50% of the patients with COVID-19. The risk of in-hospital mortality was significantly increased in subjects with hypertension and diabetes [26]. After adjusting for other risk factors, obesity in the chinese COVID-19 patients was associated with approximately 2.4-times higher risk for raising an intense pneumonia in comparison to non-obese subjects [27]. Hence, the components of MetS seem to be associated with severe COVID-19 complications.

Ghoneim et al. carried-out a population-based study to determine the relationship of MetS and its components with the risk of COVID-19 [28]. They assessed information from a large commercial database that combines electronic health records from 26 large nationwide healthcare systems. The analysis indicated that incidence of COVID-19 was higher in patients with MetS (OR = 7.00; 95% CI = 6.11–8.01). Furthermore, the adjusted OR of developing COVID-19 was increased in cases with hypertension (OR = 2.53, 95% CI = 2.40–2.68), obesity (OR = 2.20, 95% CI = 2.10–2.32), diabetes (OR = 1.41, 95% CI, 1.33–1.48), hyperlipidemia (OR = 1.70, 95% CI = 1.56–1.74). This study showed that patients with MetS and/or its each component had increased proneness to develop COVID-19. Scalsky et al. evaluated the effect of lipid profiles, BMI, and diabetes on the risk of positive test results for SARS-CoV-2 among 9,005 UK Biobank (UKBB) subjects from March 16 to June 29, 2020. Hemoglobin A1c (HbA1c), BMI, and T2DM were associated with higher risk of SARS-CoV-2 infection. However, HDL and apolipoprotein A were associated with decreased SARS-CoV-2 infection risk [29]. Our study also indicated a significant association of MetS components such as obesity, diabetes and dyslipidemia with the susceptibility to SARS-CoV-2 infection.

Among the 1000 MetS patients, we identified 206 cases with COVID-19 infection. We observed that BMI was higher significantly in the MetS cases with COVID-19 in comparison to MetS cases without COVID-19 infection. In addition, WC was significantly higher in the MetS cases with COVID-19 in comparison to MetS cases without COVID-19 infection in both males and females. Furthermore, obesity was associated with increased susceptibility to COVID-19 infection in patients with MetS (OR = 2.00, 95%CI = 1.47–2.74).

Our analysis also indicated that total cholesterol was significantly higher in the MetS cases with COVID-19 in comparison to MetS cases without COVID-19 infection. TG level was significantly higher in the MetS cases with COVID-19 compared with MetS cases without COVID-19 infection. It was detected that LDL level was significantly higher in the MetS cases with COVID-19 compared with MetS cases without COVID-19 infection. It was detected that FBS level was significantly higher in the MetS cases with COVID-19 compared with MetS cases without COVID-19 infection. However, no statistically significant difference was detected in the HDL level between MetS cases with COVID-19 compared with MetS cases without COVID-19 infection. The analysis showed that dyslipidemia was associated with an increased chance of COVID-19 infection (OR = 1.50, 95%CI = 1.10–2.05).

Among the co-morbidities in the MetS patients, we observed that CVD was associated with increased risk of COVID-19 infection in MetS patients (OR = 1.62, 95%CI = 1.09–2.40). Moreover, T2DM was associated with increased risk of COVID-19 infection in MetS patients (OR = 1.43, 95%CI = 1.01-2.00). In addition, hypertension was observed to increase the chance of COVID-19 infection in the MetS patients (OR = 1.44, 95%CI = 1.05–1.98). Furthermore, asthma (OR = 1.84, 95%CI = 1.13–2.90) and lung diseases (OR = 2.75, 95%CI = 1.93–3.92) in the MetS patients significantly increased the chance of COVID-19 infection.

During obesity-induced insulin resistance, higher levels of tumor necrosis factor (TNF)-α and interleukin (IL)-6 secreted by adipose tissue may explain the associations of coagulopathy and endothelial dysfunction with insulin resistance [30]. On the other hand, there is an association between insulin resistance and increased levels of plasminogen activator inhibitor-1 (PAI-1, which is a fibrinolytic inhibitor), and insulin and lipoproteins stimulate higher production of PAI-1 by liver and endothelial cells [30]. Higher levels of pro-inflammatory cytokines, endothelial dysfunction and a pro-coagulant condition have already been defined in obese individuals even prior to COVID-19 [31, 32]. It seems that SARS-CoV-2 infection may increase generation of inflammatory cytokines, resulting in cytokine storm, which in turn might develop thrombosis and endothelial injury. In our subjects with MetS, blood leukocytes and inflammatory indices were associated with COVID-19 infection in the MetS patients. Total number of leukocytes was observed to be significantly higher in MetS cases with COVID-19 in relation to MetS cases without COVID-19 infection. It was seen that lymphocyte-total leukocyte ratio was significantly decreased in MetS cases with COVID-19 in relation to MetS cases without COVID-19 infection. However, the neutrophil-lymphocyte ratio was significantly increased in MetS cases with COVID-19 in relation to MetS cases without COVID-19 infection. CRP level was significantly higher in MetS cases with COVID-19 in relation to MetS cases without COVID-19 infection. Additionally, ESR level was significantly higher in MetS cases with COVID-19 in relation to MetS cases without COVID-19 infection.

According to meta-analysis results [33–35], obesity, dyslipidemia, hypertension and diabetes, which are the components of MetS, are strongly involved in the aggravation of COVID-19. In addition, it was observed that CVD, which is caused by MetS, is also involved in the aggravation of COVID-19 [36]. We observed that CVD was associated with increased risk of COVID-19 infection in MetS patients (OR = 1.62, 95%CI = 1.09–2.40). Moreover, familial history of CVD was associated with increased susceptibility to COVID-19 infection in the MetS patients (OR = 1.57, 95%CI: 1.06–2.34, *P* = 0.0245).

Smoking and air pollution have been linked with upregulation of the ACE2 receptor (which is ligated with SARS-CoV-2 S protein), on adipose tissue as well as alveolar epithelial cells of lung. On the other hand. An association has been reported between air pollution and COVID-19 and smokers show more severe presentations of COVID-19. Smoking and air pollution may adversely modulate the Renin-angiotensin system (RAS) and increase the risk of COVID-19 development [37]. Our study also indicated that smoking was associated with higher chance of COVID-19 infection in the MetS patients (OR = 5.04, 95%CI = 3.53–7.21).

Even though we tried to perform a study with lowest possible confining points and biases, still some limitations and caveats exist that need to be clarified. First, the prospective design of the study might yield in limited evidence to understand underlying mechanisms involved in the modulation of COVID-19 risk in the MetS patients; hence the conclusions could only be indicative of on associations and do not represent causation. Second, a number of cases might fall in bias as to report researchers if they have COVID-19 symptoms and hence were abolished from molecular evaluations. Third, most of the patients we recruited were inhabitants of Rafsanjan city, not nationwide; therefore, we barely generalize our data to the whole country. Fourth, although we attempted to find out the contributing biological factors to alter the risk of COVID-19 infection in the MetS cases, a number of bias sources like socioeconomic situation might also be important that were not assessed in this research.

Considering all the facts, this study indicated that components of MetS, such as obesity, diabetes, dyslipidemia, cardiovascular complications were associated with increased chance of COVID-19 infection development in Iranian population and probably with aggravated symptoms in such patients.

## Data Availability

Data are available by the corresponding author upon reasonable request.

## List of abbreviations

MetS: Metabolic syndrome
COVID-19: Coronavirus disease 2019
IDF: International Diabetes Federation
SARS-CoV‐2: Severe acute respiratory syndrome coronavirus 2
ARDS: Acute respiratory distress syndrome
ACE2: Angiotensin-converting enzyme 2
WHO: World Health Organization
CVD: Cardiovascular diseases
TG: Triglyceride
HDL: High-density lipoprotein
LDL: Low-density lipoprotein
BP: Blood pressure
FBS: Fasting blood sugar
CT: Computed tomography
WC: Waist circumference
BMI: Body mass index
SPSS: Statistical Package for the Social Sciences
OR: Odds ratio
CI: Confidence interval
SD: Standard deviation
TNF: Tumor necrosis factor
PAI: Plasminogen activator inhibitor
RAS: Renin-angiotensin system
WBC: White blood cell
CRP: C-reactive protein
ALP: Alkaline phosphatase
AST: Aspartate aminotransferase
ALT: Alanine aminotransferase
LDH: Lactate dehydrogenase
ESR: Erythrocyte sedimentation rate
BUN: Blood urea nitrogen
T2DM: Type 2 diabetes mellitus
